# RETRACTED: Upfold et al. Tyrosine Kinase Inhibitors Target B Lymphocytes. *Biomolecules* 2023, *13*, 438

**DOI:** 10.3390/biom16020294

**Published:** 2026-02-13

**Authors:** Nikki Lyn Esnardo Upfold, Pavlo Petakh, Aleksandr Kamyshnyi, Valentyn Oksenych

**Affiliations:** 1 Department of Clinical and Molecular Medicine (IKOM), Norwegian University of Science and Technology (NTNU), 7028 Trondheim, Norway; 2 Department of Biochemistry and Pharmacology, Uzhhorod National University, 88000 Uzhhorod, Ukraine; 3 Department of Microbiology, Virology, and Immunology, I. Horbachevsky Ternopil National Medical University, 46001 Ternopil, Ukraine; 4 Institute of Clinical Medicine (Klinmed), University of Oslo, 0318 Oslo, Norway

The journal retracts the article titled “Tyrosine Kinase Inhibitors Target B Lymphocytes” [1], cited above.

Following publication, concerns were raised about the ownership of the data presented within this article and the accuracy of the authorship contributions.

In accordance with standard journal procedure, the Editorial Office and Editorial Board conducted an investigation with input from the relevant institutions and governing bodies, which concluded that appropriate permission to publish the data had not been obtained prior to publication, and that retroactive permission could not be obtained. The authors fully cooperated with the Editorial Office investigation; however, the Editorial Office and Editorial Board concurred with the presented findings and recommendation from the institution. As the disputed data have a substantial impact on the findings and results of the publication the Editorial Board has decided to retract this article as per MDPI’s retraction policy.

This retraction was approved by the Editor-in-Chief of *Biomolecules*.

The authors have been informed of this retraction and have indicated their disagreement with this decision.
